# Supplementary material for: The Nutritional Profile of Food Advertising for School-Aged Children via Television: A Longitudinal Approach
Source: Children (Basel). 2020 Nov 17;7(11):230. doi: 10.3390/children7110230 (PMC7698276; doi:10.3390/children7110230)
Supplement: Supplementary file 1 [file children-07-00230-s001.pdf]

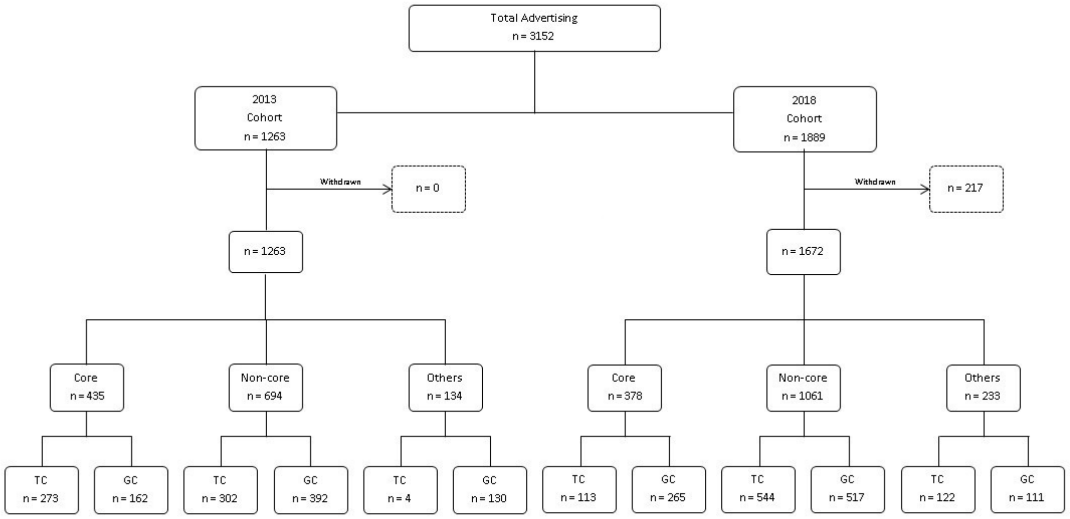

Figure S1. Number of food advertisements broadcast in 2013 and 2018.

Table S1. Encoding frames for food advertising categories and criteria for classification according to food nutritional profile advertised.

Type of Advert	Subtypes
Core Food Advertisements (CFAs) High in energy and unbalanced energy profile	Vegetables and vegetable products without added sugar
	Bottled water
	Dairy products (<3 g lipids), low-sugar and high-fiber breakfast cereals (<20 g sugar/100 g and <5 g fiber/100 g)
	Low-fat/reduced-fat milk, yoghurt, custard (<3 g fat/100 g), cheese (<15 g fat/100 g), alternatives (including probiotic drinks), infant food (excluding milk formulas)
	Fruit and fruit products without added sugar
	Meat and similar products (not breaded or battered; including fish, legumes, eggs, nuts, nut products and peanut butter, excluding sugar-coated and salted nuts)
	Soups (<2 g lipids/100 g, excluding dehydrates), salads, and sandwiches, frozen meals (<10 g lipids/portion) and low-fat savoury sauces (<10 g lipids/100 g)
	Bread (including high-fiber bread and low-fat crackers), rice, pasta and noodles
	Healthy habits (physical or mental activities related to health)
Non-core Food Advertisements (NCFAs) High in energy and unbalanced energy profile	Frozen/fried potato products (excluding chips)
	Ice cream and iced confections
	Fruit juice and fruit drinks
	Breaded or battered meat and similar products and high-fat frozen meals (>10 g lipids/100 g)
	Alcohol
	Cakes, muffins, cookies, high-fat crackers, pies, and pastries
	Sugar-sweetened drinks, including soft drinks, cordials, sports drinks and flavor additions (including diet varieties)
	Snack foods, including chips, extruded snacks, popcorn, snack and granola bars, sugar-sweetened fruit and vegetable products, and sugar-coated or salted nuts
	High-sugar or low-fiber breakfast cereals (>20 g sugar/100 g or <5 g fiber/100 g)
	Whole milk, yoghurt, custard, dairy desserts (>3 g fat/100 g), cheese and similar products
	High-fat, high-sugar, high-salt spreads (excluding peanut butter), oils and high-fat savoury sauces
	Chocolate and confectionery (including regular and sugar-free chewing gum and sugar)
	Fast-food restaurants and meals (including "healthy" alternatives)
Other Food Advertisements (OFAs) Special food such as vitamins, supplements and baby formulas, and supermarkets	Baby and toddler milk formulas
	Vitamin and mineral supplements
	Supermarkets that advertise mostly core foods
	Tea and coffee
	Supermarkets that advertise mostly non-core foods
	Supermarkets with no specified food (generic advertisements or not clearly for core or non-core foods)

Table S2. Relative risk of seeing non-core food adverts on children's TV channels in 2018 vs. 2013.

NCFA Category	R.R.	C.I. 95%		χ^2 correct	d.f.	*p*-Value
		Lower	Upper			
Ice cream and iced confections	0.018	0.002	0.128	54.999	1	<0.001
Breaded or battered meat and similar products and high-fat frozen meals	3.504	1.561	7.864	10.647	1	<0.001
Cakes, muffins, cookies, high-fat crackers, pies and pastries	29.730	4.099	215.631	27.930	1	<0.001
Snack foods, including chips, extruded snacks, popcorn, snack and granola bars, sugar-sweetened fruit and vegetable products, and sugar-coated or salted nuts	1.009	0.510	1.995	0.001	1	ns
High-sugar or low-fiber breakfast cereals	2.091	1.585	2.758	29.707	1	<0.001
Whole milk, yoghurt, custard, dairy desserts, cheese and similar products	0.831	0.677	1.020	3.143	1	ns
Chocolate and confectionery	16.054	6.592	39.099	73.583	1	<0.001
Fast-food restaurants or meals	0.773	0.451	1.324	0.883	1	ns

NCFA, non-core food advertisement; RR, relative risk; CI, confidence interval; χ^2 correct, adjusted chi-squared; d.f., degree of freedom; ns, non-significant. Significance level for statistical tests: *p*-value < 0.05.

Table S3. Odds ratios of exposure to non-core food adverts on children's and general TV channels in 2018.

Non-Core Food Adverts	OR	C.I. 95%		χ^2 correct	d.f.	*p*-Value
		Lower	Upper			
Breaded or battered meat and similar products and high-fat frozen meals	3.906	1.912	7.978	16.126	1	<0.001
Cakes, muffins, cookies, high-fat crackers, pies and pastries	3.664	1.945	6.902	18.347	1	<0.001
Snack foods, including chips, extruded snacks, popcorn, snack and granola bars, sugar-sweetened fruit and vegetable products, and sugar-coated or salted nuts	0.834	0.458	1.518	0.355	1	ns
High-sugar or low-fiber breakfast cereals	2.574	1.942	3.412	45.337	1	<0.001
Whole milk, yoghurt, custard, dairy desserts, cheese and similar products	2.764	2.050	3.728	47.009	1	<0.001
Chocolate and confectionery	0.830	0.634	1.088	1.825	1	ns
Fast-food restaurants or meals	0.260	0.166	0.407	39.185	1	<0.001

OR, odds ratio; CI, confidence interval; χ^2 correct, adjusted chi-squared; d.f., degree of freedom; ns, non-significant. Significance level for statistical tests: p-value < 0.05.
